# ChatGPT and scientific papers in veterinary neurology; is the genie out of the bottle?

**DOI:** 10.3389/fvets.2023.1272755

**Published:** 2023-10-05

**Authors:** Samira Abani, Holger Andreas Volk, Steven De Decker, Joe Fenn, Clare Rusbridge, Marios Charalambous, Rita Goncalves, Rodrigo Gutierrez-Quintana, Shenja Loderstedt, Thomas Flegel, Carlos Ros, Thilo von Klopmann, Henning Christian Schenk, Marion Kornberg, Nina Meyerhoff, Andrea Tipold, Jasmin Nicole Nessler

**Affiliations:** ^1^Department of Small Animal Medicine and Surgery, University of Veterinary Medicine Hannover, Hannover, Germany; ^2^Centre for Systems Neuroscience, University of Veterinary Medicine Hannover, Hannover, Germany; ^3^Department of Veterinary Clinical Science and Services, Royal Veterinary College, University of London, London, United Kingdom; ^4^Faculty of Health and Medical Sciences, School of Veterinary Medicine, University of Surrey, Guildford, United Kingdom; ^5^Department of Veterinary Science, Small Animal Teaching Hospital, University of Liverpool, Neston, United Kingdom; ^6^Small Animal Hospital, School of Biodiversity, One Health and Veterinary Medicine, University of Glasgow, Glasgow, United Kingdom; ^7^Department of Small Animal Medicine, Leipzig University, Leipzig, Germany; ^8^Memvet Referral Veterinary Center, Palma de Mallorca, Spain; ^9^Department of Neurology, Small Animal Clinic Hofheim, Hofheim, Germany; ^10^Department of Neurology, Lüneburg Small Animal Clinic, Lüneburg, Germany; ^11^AniCura Small Animal Clinic, Trier, Germany

**Keywords:** ChatGPT, artificial intelligence (AI), machine learning, generative AI, scientific writing, ethics, integrity, plagiarism

## Introduction

The new wave of technology known as generative artificial intelligence (AI), while both awe-inspiring and intimidating due to its transformative potential, has become a worldwide phenomenon this year (1). Generative AI refers to a class of AI models that can create new content, such as text, images, sounds, and videos, based on patterns and structures learned from existing data (2, 3).

Since its launch in November 2022, ChatGPT^®^ (OpenAI^®^, L.L.C., San Francisco, CA, USA), a state-of-the-art language model, has taken the world by storm and captured the attention of the scientific community (GPT stands for “generative pretrained transformer”) (4, 5). The model is trained to predict the next word in a sentence using hundreds of gigabytes of online textual data and has been further fine-tuned with both supervised and reinforcement learning techniques (6, 7). Although models like ChatGPT^®^ can generate highly plausible and sometimes remarkably coherent text in a wide range of contexts, they rely solely on statistical representations of language and lack any understanding of the meanings behind their generated output (8).

Many challenges and concerns arise regarding the dual-use nature of Large Language Models (LLMs), such as ChatGPT^®^, particularly in scholarly publishing (9). While some believe that journals must take action against such tools to battle the flood of AI-generated manuscripts that could potentially ruin the scholarly publishing industry, others argue that these technologies will break down language barriers and increase global participation in scholarly conversations (10, 11).

As AI chatbots become more integrated into our daily lives, it is predictable that the way internet users find information online will change (12). However, while using AI chatbots makes finding information faster and simpler compared to traditional search methods, there is still a tendency for such models to “hallucinate” and create inaccurate fictional answers, which can mislead individuals (12, 13). Moreover, unlike search engines, ChatGPT^®^ engages users in conversation or provides a concise answer, rather than directing them to numerous websites that offer a plethora of information from various sources and letting users decide what they trust. Additionally, ChatGPT^®^ (free version, June 2023) does not browse the web to gather real-time information; instead, its knowledge is restricted to the limited dataset it learned until 2021 (5). Consequently, its responses may not reflect the most recent information and are constrained by the content it was trained on OpenAI (5). LLMs like ChatGPT^®^ have applications far beyond search engines. Among others, ChatGPT^®^ is even capable of generating a credible scientific manuscript when provided with the appropriate prompt. Surprisingly, the AI-generated papers were “convincing enough” to initiate debates in academic community concerning the use of such tools in scholarly publishing (14).

Almost everywhere in the research community, the disruptive potential of AI tools is being eagerly debated. There are some observers who worry that in the worst-case scenario AI-generated technologies will increase the output of “pseudo-scientific papers” even faster and cheaper (15, 16). Another major concern related to the utilization of LLMs like ChatGPT^®^ in scientific writing is so called “AI-based plagiarism” or “copyright laundering” (17, 18). Chan (17), define it as *AI-giarism*, referring to “the unethical use of AI to create content that is plagiarized from either original human-authored work or directly from AI-generated content, without appropriately acknowledging the original sources or AI's contribution.” Concerning this, shortly after the launch of ChatGPT^®^, some publishers established new policies regarding the disclosure of such tools, while others have taken a step further and announced a complete ban on text generated by ChatGPT^®^ or any other AI tools (19). However, it remains uncertain whether there is currently an enforcement tool capable of consistently detecting AI-generated text (15).

While there has been a rapidly growing academic literature investigating the potential and limitations of ChatGPT^®^ across diverse domains, there is no published work (as of June 2023) on the potential uses and misuses of ChatGPT^®^ and its ethical boundaries in the context of scientific writing within the field of veterinary medicine. First and foremost, this opinion article reflects the authors' viewpoints and experiences rather than providing a comprehensive, critical review. In the current study, we present a user-based experience to determine if ChatGPT^®^ can generate convincible scientific papers in the field of veterinary neurology. We evaluated the ChatGPT^®^ generated abstracts as well as introduction sections with references for three research papers focusing on different subjects in veterinary neurology. We used an AI output detector and a plagiarism detector to analyse the generated content. Furthermore, we asked thirteen Board-Certified neurologists by the European College of Veterinary Neurology or American College of Veterinary Internal Medicine (subspecialty of Neurology), who have experience in writing and reading scientific papers, to try to evaluate whether the sets were original or AI-generated. The purpose of this study is to focus on the potential limitations and advantages associated with the application of ChatGPT^®^ in scientific writing within the field of veterinary neurology.

## Materials and methods

We selected three research papers authored by the team of the Department of Small Animal Medicine and Surgery at the University of Veterinary Medicine Hannover, Hannover, Germany focusing on different subjects in the field of veterinary neurology, including SARS-CoV-2 scent detection in dogs (20), potential biomarkers for steroid-responsive meningitis-arteritis (21), and staining of cannabinoid receptor type one (22). These articles were published between November 2022 and April 2023 in three different journals: BMJ (23), PLOS ONE (24), and Scientific Reports (25). As stated on the OpenAI^®^ homepage, ChatGPT^®^ does not have access to data beyond 2021 (26). Therefore, we assume that the model has not accessed any of these publications. Given the diverse research interests and familiarity levels of the specialist reviewers with the subjects, the three subjects were classified into three familiarity classes:

In Test 1, the subject of SARS-CoV-2 scent detection in dogs was categorized as less familiar, assuming limited familiarity by the reviewers with this topic.

In Test 2, the subject of potential biomarkers for steroid-responsive meningitis-arteritis in dogs was classified as highly familiar.

Lastly, in Test 3, the staining of cannabinoid receptor type one was classified as a moderately familiar subject.

We hypothesized that reviewers might show different levels of ability in distinguishing between AI-written manuscripts and human-written ones, depending on their familiarity with the subject or research interest. We utilized two different plagiarism detection tools to analyse both the original and AI-generated manuscripts. The first tool used is a paid Internet-based similarity detection service called Turnitin^®^ (27), offering a similarity index ranging from 0 to 100 percent. A higher value on Turnitin indicates a greater level of text redundancy with existing sources. The second tool used is a free online plagiarism checker platform called Plagiarism Detector (28), which provides a plagiarized score ranging from 0 to 100 percent. A higher plagiarized score indicates more detected plagiarism. In addition, we evaluated all the original and generated manuscripts using two different AI-generated content detectors. The first AI-detector is called the AI Detector (29), which provides scores indicating the percentage of human-written content. A score of 100% indicates the absence of any detected AI-written content. The second AI-detector is known as the AI Text Classifier (OpenAI^®^) (30), which categorizes contents as either very unlikely, unlikely, unclear, possible, or likely to be AI-generated.

### Generating the abstract and introduction with references

We utilized ChatGPT^®^ to generate abstracts, introductions, and references for three mentioned scientific papers based on the title, keywords, journal style and characteristics of each author.

### Prompts

To generate scientific abstracts and introduction sections with references, we used different prompts. We prompted ChatGPT^®^ with following request “Write an academic abstract with a focus on (subject) in the style of (author characteristics, i.e., position, gender and age) at (university name) for publication in (journal name), (keywords).” Additionally, during a separate chat session, we asked “Write an introduction on (subject), including the following keywords ….” Subsequently, we requested “Generate 15 references to support the content.”

During our interactions with ChatGPT^®^ for generating scientific papers (May 2023), we noticed that the model sometimes disobeyed the requests. For example, when we prompted it to generate references to support the content, the model answered: “As an AI language model, I don't have direct access to a specific database or the ability to provide real-time references. I can provide general information and knowledge based on what has been trained on. For accurate and up-to-date references, it's recommended to consult academic databases, research papers, and scholarly sources.” In contrast, when the prompt was changed to “Generate 15 fictitious references to support the content” the model generated 15 fictitious references and responded with the following sentence: “Please note that these references are fictional and not based on real publications.” Although it feels rather odd to be lectured by an AI model, we acknowledge that the model demonstrates some knowledge of producing fake or inaccurate data. In addition, ChatGPT^®^'s generated responses are sensitive to the way it is prompted, and it can generate different responses even for the same prompt multiple times (31). To enhance the believability and persuasiveness of the responses generated by ChatGPT^®^, we prompted it multiple times with additional refinements, such as font and reference styles, within the generated text.

### Evaluation

We requested thirteen expert reviewers, all Diplomates of the European College of Veterinary Neurology or American College of Veterinary Internal Medicine (SDD, JF, ClR, MC, RG, RGQ, SL, TF, CaR, TK, HS, MK, NM), to evaluate three tests; all three tests are available in the Supplementary material. Each test contained two sets of abstracts and introductions with references, with one set being written by ChatGPT^®^ and the other by a veterinary neurologist. The reviewers were asked to indicate whether the manuscripts were written by human, generated by an AI algorithm, or if they were unable to determine the source. All specialist reviewers were blinded to the information who generated the text they evaluated. All manuscript headings, as well as any references containing the names of authors or reviewers involved in this current study, were deleted, to avoid bias in the evaluation. To ensure the accuracy of our study, we requested that reviewers avoid opening all three tests simultaneously for comparison. Additionally, we asked them not to use the internet, AI output detector software, or any external sources such as books or scientific papers during the survey. The process for each of the three tests consisted of the following stages:

Initial assessment stage: abstract identification. The reviewer was requested to read both abstracts and determine which one was written by AI, without referring to the introduction or references.

Final assessment stage: introduction and reference review. The reviewer was asked to read the introduction and references for the corresponding abstract. Considering all the information provided (abstract, introduction, and references), the reviewers were asked to select the manuscript they thought was written by an AI, and to provide supporting evidence and an explanation for their choice.

## Results

**Figure 1:**
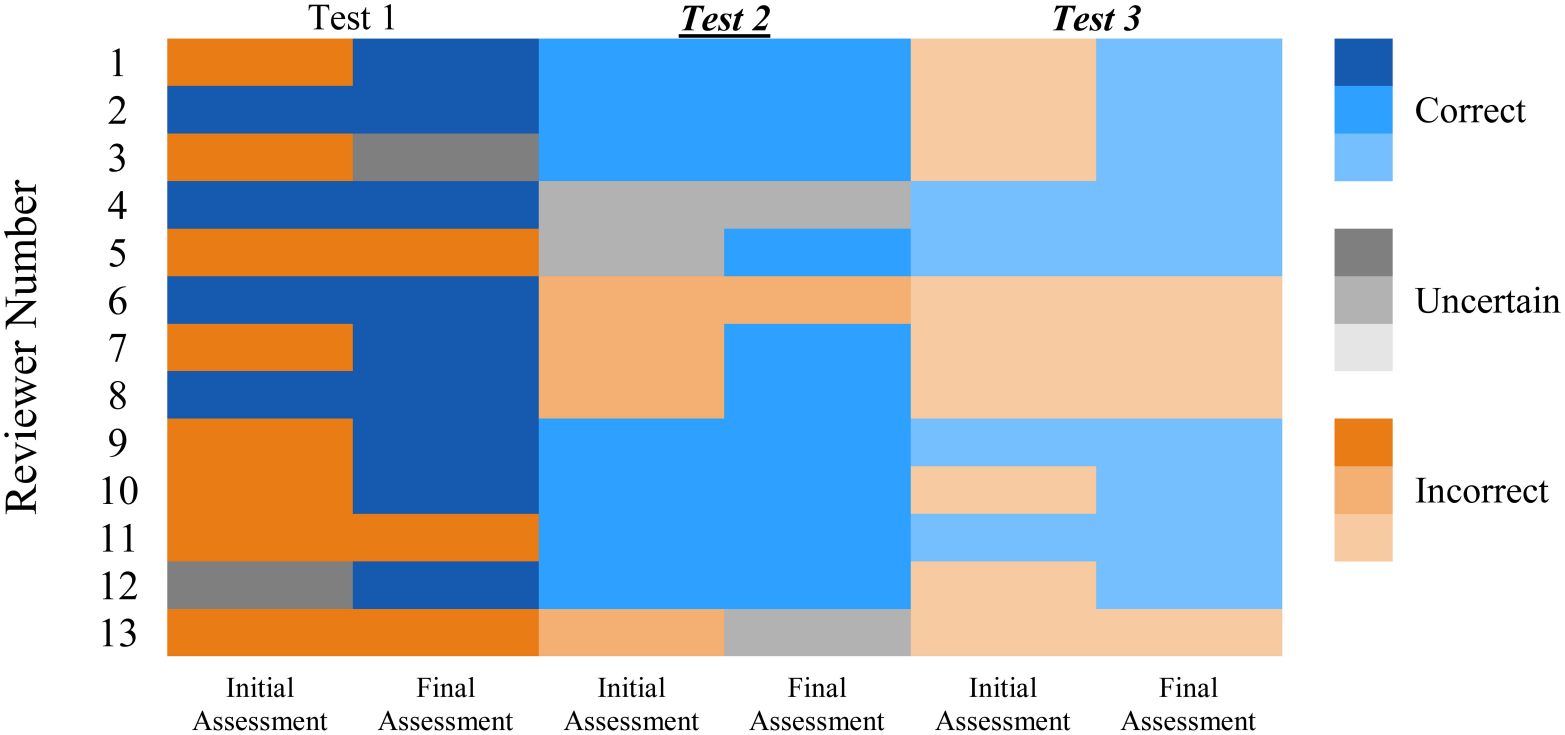
Presents the performance of specialist reviewers in identifying ChatGPT^®^-generated scientific manuscripts vs. original published manuscripts in three tests, each containing two stages; the initial assessment, referred to as abstract detection, and the final assessment, which includes the introduction with references. The reviewers' assessments are represented using colors, with red indicating “Incorrect” (misidentification), green indicating “Correct” (accurate identification), and yellow indicating “Uncertain” (indicating uncertainty). Reviewers demonstrated a better performance in identifying ChatGPT^®^-generated manuscripts in **Test 2** (the highly familiar subject of steroid-responsive meningitis-arteritis in dogs) vs. *Test 1* (SARS-CoV-2 detection dogs) and **Test 3** (Cannabinoid receptors in canine epilepsy), which were referred to as less familiar and moderately familiar subjects. In the final assessment, reviewers demonstrated improved performance in three tests, indicating that the reviewers were able to refine their assessments and make more accurate conclusions by incorporating specific information and references.

In Test 1 and Test 3, which were referred to as less familiar and moderately familiar subjects, respectively, only four out of 13 specialist reviewers (31%) correctly identified the AI-generated abstracts (initial assessment stage). In the final assessment stage of both Test 1 and Test 3, which included the introduction and references, nine out of 13 reviewers (69%) successfully identified the generated abstracts and introductions. In Test 1, four reviewers, and in Test 3, five reviewers, who initially could not identify the AI-generated abstracts, identified the manuscripts as being generated by the AI algorithm after reading the introduction and references. In the initial assessment stage of Test 2, which was classified as a highly familiar subject in the field of veterinary neurology, seven out of 13 reviewers (54%) were able to correctly identify the AI-generated abstracts. In the final assessment stage, which included the introduction and references, 10 out of 13 participants (77%) correctly identified the AI-generated manuscript. Furthermore, five reviewers, who initially misidentified the human-written abstract as AI-generated in Test 2, re-evaluated their decision after reading the introduction and references. They subsequently correctly identified the manuscript as being written by a human (Figure 1).

Specialist reviewers provided various explanations to determine whether the manuscript was generated by AI or written by a person. They commented that AI-generated manuscripts sometimes contain “incredibly human-sounding” texts and might have better English proficiency and structure. As a result, it was occasionally impossible to differentiate the AI-generated manuscripts from the original ones without relying on scientific background information and references. However, some reviewers also pointed out that AI-generated manuscripts were verbose, superficial, and less creative compared to human-written ones.

In addition, both the original and AI-generated manuscripts, were analyzed using the paid Internet-based similarity detection service called Turnitin^®^ (27) and the free online plagiarism checker website called Plagiarism Detector (28). When using Turnitin^®^, it indicated a similarity index of 100 (higher value indicates more similar text) for all original published manuscripts in Test 1, Test 2, and Test 3. In comparison, the AI-generated manuscripts in Test 1, Test 2, and Test 3 had similarity indices of 2%, 8%, and 18%, respectively, on Turnitin^®^. When utilizing the free online plagiarism checker website, it indicated plagiarism scores of 62%, 74%, and 58% for the original manuscripts in Test 1, Test 2, and Test 3, respectively. In contrast, the website detected 0% plagiarized content in the AI-generated manuscripts of Test 1, Test 2, and Test 3, indicating the potential absence of any detected plagiarism.

When the original manuscripts were run through the online platform AI Detector (29), it indicated that all the original manuscripts contained 0% AI-written content. Among the AI-generated manuscripts, the AI Detector identified AI-generated manuscript in Test 2, indicating that 100% of the content was written by an AI algorithm. However, it identified two other AI-generated manuscripts in Test 1 and Test 3 as having 0% content written by an AI algorithm. The AI Text Classifier (30) indicated that the original manuscript in Test 1 unlikely, in Test 2 and Test 3 was very unlikely to being generated by an AI algorithm. Nonetheless, the AI Text Classifier correctly identified all the abstracts and introductions generated by ChatGPT^®^ as possibly AI-generated.

## Discussion

The present study showed that experts in the field increasingly struggled to distinguish between ChatGPT^®^- and human-written abstracts with a decrease in subject knowledge. In the subject matter which was least familiar, only four out of 13 reviewers correctly identified the text written by AI, whereas when the topic was more familiar, this rate increased to around half, with seven out of 13 reviewers correctly identifying the AI-written abstract. The expert reviewers did, however, show improvement in accurately detecting the text when they had access to additional sections, including the introduction and reference list. This led to an increase in their performance, with approximately two out of three texts being correctly identified. A software tool, AI Text Classifier (OpenAI^®^), on the other hand, correctly identified the generated texts. The reviewers commented on different factors to judge whether a manuscript was written by AI or a human, sometimes relying on the same aspect of the text to reach contrasting conclusions. Hence, human reviewers can be tricked by sophisticated AI-generated articles, as LLMs like ChatGPT^®^ are trained to mimic human writing styles and produce coherent, plausible sounding texts. Furthermore, scientific manuscripts may sometimes present poor grammar or readability, when written by humans. Ultimately, relying solely on naturalness, fluency and writing patterns may not always assist reviewers in distinguishing between content written by humans and content generated by AI (32). Specialists comments which highlights this were:

“*The English in manuscript B [AI-generated] is better….” [Reviewer 11, Test 3]*

“*I found this [Test 1] difficult. I think abstract B [human written] is worded in a slightly more robotic manner, although this could just be concise wording.” [Reviewer 2, Test 1]*

On the other hand, the inclusion of the introduction, along with references, improved the reviewers' ability to differentiate between human-written and AI-generated articles. Many reviewers' comments focused on the textual content and emphasized that the detailed information helped them judge whether the texts were original or contained fabricated information.

“*My only reason to select abstract A [AI-generated] as written by AI is the ‘generic references'; Davis, Johnson, and Smith can appear a bit random, the reference list comes across as potentially legit.” [Reviewer 10, Test 3]*

Language models, like ChatGPT^®^, could generate convincing scientific manuscripts with fabricated data that might also include fictitious or inaccurate information (see the generated manuscripts in Supplementary material).

Despite recent improvements, LLMs like ChatGPT^®^ are still prone to “hallucination.” Thus, this raises concerns regarding the integrity of utilizing such models in academic writing. Moreover, plagiarism detection tools may not flag AI-generated manuscripts. Since ChatGPT^®^ is trained on vast datasets from the internet, some content may include the intellectual property of authors without explicit permission or proper citation (17). Consequently, AI-generative models might create new texts that resemble the original works without acknowledgment, leading to “AI-giarism.” Furthermore, reviewers demonstrated varying levels of confidence and success in distinguishing between AI-written manuscripts and the original published work, depending on their level of subject knowledge. Specialist reviewers found Test 1, which we referred to as the less familiar subject, and Test 3, which we referred to as a moderately familiar subject, more challenging due to their unfamiliarity with these subjects.

“*I struggled more with the COVID abstracts [SARS-CoV-2 scent detection in dogs] as it is not a topic I know much about…” [Reviewer 9, Test 1]*

“*The references were difficult to assess: the specific topic [staining of cannabinoid receptor type one] falls outside my personal field of research interest and seem to originate all from more ‘basic science' journals.” [Reviewer 10, Test 3]*

In contrast, for Test 2, which focused on the highly familiar subject area of steroid-responsive meningitis-arteritis in dogs, specialist reviewers showed better performance in identifying the ChatGPT^®^-generated manuscript. In conclusion, specialist reviewers mentioned that when the manuscript was related to their research field, it was easier to distinguish between human-written and AI-written texts.

“*In Abstract B [AI-generated]—SRMA can cause severe neurological deficits—but that is rare. SRMA is only life-threatening if not treated. I don't think a neurologist would write this sentence as the first introductory sentence.” [Reviewer 11, Test 2]*

It seems that AI-based generative tools have already touched the scientific publishing flow and as they continue to improve, they will create opportunities, as well as threats in every aspect of science in the future (33). We believe that AI- models, if trained and tested properly on diverse, reliable, and representative datasets, with a focus on transparency and accuracy, could serve as valuable tools for a wide range of applications in research and publishing. These include AI-tools to assist with semantic search, writing, editing, summarizing papers, statistical analysis, submission screening, citation validation, and peer reviewing (33, 34). In the age of globalization, diversity, equity, and inclusivity bring new ideas, dynamics, and creativity to every domain, and science is not an exception. However, English is a dominant or “standard” language in science, thus can create language barriers for non-native English-speaking researchers who are enthusiastic about contributing to scholarly publishing. Undeniably, publishing in the world's influential scientific journals requires the privilege of being a so-called “native English speaker.” AI tools like ChatGPT^®^ have the potential to address the often neglected challenge of the language barrier in science, offering valuable assistance to non-native English speaker researchers and facilitating the transfer of scientific knowledge in both directions. Some researchers already utilize AI tools such as Grammarly^®^ (35), Paperpal^®^ (36), Hemingway Editor (37), and Writefull (38) to assist in editing and improving their manuscripts, aiming to increase readability, clarity, and correctness (34, 39).

Language models might become a “game-changer” in scientific writing, enabling researchers to use these models as tools to translate knowledge into their language or improve their English scientific writing skills. This could ultimately be advantageous not only for the researchers but also for the other side of scholarly literature—journals, by reducing the rejection of scientific papers due to poor English.

In the context of the implications of AI technology on scientific writing, journals defined different policies. Some publishers, such as Frontiers (40) and Springer Nature^®^ (41), state that AI writing tools like ChatGPT^®^ cannot be credited as authors. However, researchers are allowed to use such tools if their usage is appropriately documented in relevant sections.

## Limitations

The current study's findings should be interpreted with caution due to limitations in study design. The reviewers might have read the original papers before the survey was conducted and might have known which abstracts were written by humans. In addition, reviewers knew that each test contained two manuscripts, with one set written by an AI-algorithm and the other by a human. As a result, in real-world scenarios, journal reviewers might show different performance in identifying AI-generated manuscripts. These might be less. Moreover, there are numerous language models available for streamlining scientific writing. However, for this study, we chose to use ChatGPT^®^ due to its rapidly growing popularity, as well as its free and user-friendly platform that can be utilized by individuals without requiring any technical expertise (42).

## Conclusion

Although this study initiates and opens a conversation regarding the utilization of language models like ChatGPT^®^ in the scientific publishing flow in veterinary neurology, the use of AI technology in scientific practice remains debatable in academia. While there is an accurate concern regarding the misuse of AI-based tools in scientific publishing, only simply setting strict boundaries may not always be the most effective way to prevent malpractice. LLMs can either pose a threat to science integrity and transparency or assist researchers, depending on how they are used. Therefore, we suggest integrating education on both proper use and potential misuse of AI-based tools in academia, as part of good scientific practice for both pre- and post-graduate students in university programs. These training programs could effectively raise awareness and address the ethical considerations associated with AI models in research integrity. The growing popularity of AI requires interdisciplinary scientific collaboration to establish clear and comprehensive guidelines and recommendations for the utilization of AI tools by publishers and journals, thus ensuring the integrity and transparency of the published literature.

## Author contributions

SA: Conceptualization, Data curation, Methodology, Writing—original draft, Formal analysis, Software, Visualization. HV: Conceptualization, Funding acquisition, Methodology, Project administration, Supervision, Writing—review and editing. SD: Writing—review and editing. JF: Writing—review and editing. CRu: Writing—review and editing. MC: Writing—review and editing. RG: Writing—review and editing. RG-Q: Writing—review and editing. SL: Writing—review and editing. TF: Writing—review and editing. CRo: Writing—review and editing. TK: Writing—review and editing. HS: Writing—review and editing. MK: Writing—review and editing. NM: Writing—review and editing. AT: Conceptualization, Methodology, Supervision, Writing—review and editing. JN: Conceptualization, Funding acquisition, Methodology, Project administration, Supervision, Writing—review and editing.

## References

[1] Wodecki B. UBS: ChatGPT May Be the Fastest Growing App of All Time. (*2023*). Available online at: https://aibusiness.com/nlp/ubs-chatgpt-is-the-fastest-growing-app-of-all-time (accessed August 1, 2023).

[2] Callaway E. How generative AI is building better antibodies. Nature. (2023) 617:235. 10.1038/d41586-023-01516-w 37142726

[3] *Generative Models*. Available online at: https://openai.com/research/generative-models (accessed July 21, 2023).

[4] Huang K. Alarmed by A.I. Chatbots, Universities Start Revamping How They Teach. The New York Times. (2023). Available online at: nytimes.com (accessed July 7, 2023).

[5] OpenAI. Available online at: https://www.nytimes.com/2023/01/16/technology/chatgpt-artificial-intelligence-universities.html (accessed July 7, 2023).

[6] Mearian L. Q&A: ChatGPT isn't Sentient, it's a Next-word Prediction Engine. Available online at: https://www.computerworld.com/article/3688934/chatgpt-is-not-sentient-it-s-a-next-word-prediction-engine.html (accessed July 7, 2023).

[7] Rudolf J Tan S Tan S. ChatGPT: bullshit spewer or the end of traditional assessments in higher education? J Appl Learn Teach. (2023) 6. 10.37074/jalt.2023.6.1.9

[8] Spitale G Biller-Andorno N Germani F. AI model GPT-3 (dis)informs us better than humans. Sci Adv. (2023) 9:eadh1850. 10.1126/sciadv.adh1850 37379395 PMC10306283

[9] Van Dis EAM Bollen J Van Rooij R Zuidema W Bockting CL. ChatGPT: five priorities for research. Nature. (2023) 614:224–6. 10.1038/d41586-023-00288-7 36737653

[10] Thorp HH. ChatGPT is fun, but not an author. Science. (2023) 379:313. 10.1126/science.adg7879 36701446

[11] Berdejo-Espinola V Amano T. AI tools can improve equity in science. Science. (2023) 379:991. 10.1126/science.adg9714 36893248

[12] Stokel-Walker C. AI chatbots are coming to search engines — can you trust the results? Nature. (2023). 10.1038/d41586-023-00423-4 36781968

[13] Sallam M. ChatGPT utility in healthcare education, research, and practice: systematic review on the promising perspectives and valid concerns. Healthcare. (2023) 11:887. 10.3390/healthcare11060887 36981544 PMC10048148

[14] Quach K. Scientists Tricked into Believing Fake Abstracts written by ChatGPT were Real. (2023). Available online at: https://www.theregister.com/2023/01/11/scientists_chatgpt_papers/?ref=allganize-korea (accessed July 7, 2023).

[15] Berinard J. Fake scientific papers are alarmingly common. Science. (2023) 380:568–69. 10.1126/science.adi652337167379

[16] Tay A. AI Writing Tools Promise Faster Manuscripts for Researchers. (2021). Available online at: https://www.nature.com/nature-index/news/artificial-intelligence-writing-tools-promise-faster-manuscripts-for-researchers (accessed July 7, 2023).

[17] Chan CKY. Is AI Changing the Rules of Academic Misconduct? An In-Depth Look at Students' Perceptions of “AI-Giarism”. (2023). 10.48550/arXiv.2306.03358

[18] Hern A. AI Bot ChatGPT Stuns Academics with Essay-writing Skills Usability. (2022). Available online at: https://www.theguardian.com/technology/2022/dec/04/ai-bot-chatgpt-stuns-academics-with-essay-writing-skills-and-usability (accessed July 21, 2023).

[19] Tools such as ChatGPT threaten transparent science; here are our ground rules for their use. Nature. (2023) 613. 10.1038/d41586-023-00191-1 36694020

[20] Ten Hagen NA Twele F Meller S Wijnen L Schulz C Schoneberg C. Canine real-time detection of SARS-CoV-2 infections in the context of a mass screening event. BMJ Glob Health. (2022) 7:e010276. 10.1136/bmjgh-2022-010276 36368765 PMC9659709

[21] Nessler JN Tipold A. Immunoglobulin profiling with large high-density peptide microarrays as screening method to detect candidate proteins for future biomarker detection in dogs with steroid-responsive meningitis-arteritis. PLoS ONE. (2023) 18:e0284010. 10.1371/journal.pone.0284010 37036858 PMC10085023

[22] Kostic D Nowakowska M Freundt Revilla J Attig F Rohn K Gualtieri F. Hippocampal expression of the cannabinoid receptor type 1 in canine epilepsy. Sci Rep. (2023) 13:3138. 10.1038/s41598-023-29868-3 36823232 PMC9950490

[23] *BMJ Journal*. Available online at: https://journals.bmj.com/home (accessed July 21, 2023).

[24] *PLOS ONE*. Available online at: https://journals.plos.org/plosone/ (accessed July 21, 2023).

[25] *Scientific Reports*. Available online at: https://www.nature.com/srep/ (accessed July 21, 2023).

[26] Raf. Do the OpenAI API Models have Knowledge of Current Events? Available online at: https://help.openai.com/en/articles/6639781-do-the-openai-api-models-have-knowledge-of-current-events (accessed July 7, 2023).

[27] Turnitin. Available online at: https://www.turnitin.com/ (accessed July 21, 2023).

[28] *Plagiarism Detector* Available online at: https://plagiarismdetector.net/ (accessed July 21, 2023).

[29] *AI Detector*. Available online at: https://plagiarismdetector.net/ai-content-detector (accessed July 21, 2023).

[30] AI Classifier. New AI Classifier for Indicating AI-written text. Available online at: https://openai.com/blog/new-ai-classifier-for-indicating-ai-written-text (accessed July 21, 2023).

[31] Gao CA Howard FM Markov NS Dyer EC Ramesh S Luo Y. Comparing scientific abstracts generated by ChatGPT to real abstracts with detectors and blinded human reviewers. npj Digit Med. (2023) 6:75. 10.1038/s41746-023-00819-6 37100871 PMC10133283

[32] Clark E August T Serrano S Haduong N Gururangan S Smith NA. Human Evaluation of Generated Text. Available online at: www.nltk.org/ (accessed July 21, 2023).

[33] Flanagin A Bibbins-Domingo K Berkwits M Christiansen SL. Nonhuman “authors” and implications for the integrity of scientific publication and medical knowledge. JAMA. (2023) 329:637–9. 10.1001/jama.2023.1344 36719674

[34] De Waard A. Guest post – AI and scholarly publishing: a view from three experts. (2023). Available online at: https://scholarlykitchen.sspnet.org/2023/01/18/guest-post-ai-and-scholarly-publishing-a-view-from-three-experts/ (accessed July 7, 2023).

[35] Grammarly. Available online at: https://www.grammarly.com/ (accessed July 21, 2023).

[36] Paperpal. Available online at: https://paperpal.com/ (accessed July 21, 2023).

[37] *Hemingway Editor*. Available online at: https://hemingwayapp.com/ (accessed July 21, 2023).

[38] Writefull. Available online at: https://www.writefull.com/ (accessed July 21, 2023).

[39] Overcoming the language barrier in science communication. Nat Rev Bioeng. (2023) 1:305. 10.1038/s44222-023-00073-1

[40] *Author, Guidelines - AI Use by Authors*. Available online at: https://www.frontiersin.org/guidelines/author-guidelines (accessed July 21, 2023).

[41] *AI Authorship*. Available online at: https://www.springer.com/jp/editorial-policies/artificial-intelligence–ai-/25428500#:~:text=While%20legal%20issues%20relating%20to%20AI-generated%20images%20and,have%20created%20images%20in%20a%20legally%20acceptable%20manner (accessed July 21, 2023).

[42] Chow AR. How ChatGPT Managed to Grow Faster Than TikTok or Instagram 4:40 PM EST. (2023). Available online at: https://time.com/6253615/chatgpt-fastest-growing/ (accessed June 1, 2023).

