# Supplementary material for: ChatGPT and scientific papers in veterinary neurology; is the genie out of the bottle?
Source: Front Vet Sci. 2023 Oct 5;10:1272755. doi: 10.3389/fvets.2023.1272755 (PMC10585059; doi:10.3389/fvets.2023.1272755)
Supplement: Supplementary file 1 [file Data_Sheet_1.DOCX]

Supplementary Material

ChatGPT and Scientific Papers in Veterinary Neurology; Is the Genie Out of the Bottle?

Samira Abani^1,2^, Holger A. Volk^1,2^, Steven De Decker^3^, Joe Fenn^3^, Clare Rusbridge^4^, Marios Charalambous^1^, Rita Goncalves^5^, Rodrigo Gutierrez-Quintana^6^, Shenja Loderstedt^7^, Thomas Flegel^7^, Carlos Ros^8^, Thilo von Klopmann^9^, Henning C. Schenk^10^, Marion Kornberg^11^, Nina Meyerhoff^1^, Andrea Tipold^1,2^, Jasmin Nessler^1*^

*Correspondence: Jasmin Nessler
[jasmin.nessler@tiho-hannover.de](mailto:jasmin.nessler@tiho-hannover.de)

^1^Department of Small Animal Medicine and Surgery, University of Veterinary Medicine Hannover, Hannover, Germany

^2^Centre for Systems Neuroscience, University of Veterinary Medicine Hannover, Hannover, Germany

^3^Department of Clinical Science and Services, Royal Veterinary College, London, United Kingdom

^4^School of Veterinary Medicine, Faculty of Health and Medical Sciences, University of Surrey, Guildford, Surrey, United Kingdom

^5^Department of Veterinary Science, Small Animal Teaching Hospital, University of Liverpool, Neston, United Kingdom

^6^Small Animal Hospital, School of Biodiversity, One Health and Veterinary Medicine, University of Glasgow, Glasgow, United Kingdom

^7^Department of Small Animal Medicine, Leipzig University, Leipzig, Germany

^8^Memvet Referral Veterinary Center, Palma de Mallorca, Balearic Islands, Spain

^9^Hofheim Small Animal Clinic, Hofheim, Germany

^10^Lüneburg Small Animal Clinic, Lüneburg, Germany

^11^AniCura Small Animal Clinic, Trier, Germany

**TEST 1**

**Task 1: Read the Abstracts**

Please carefully read two abstracts labeled as Abstract A and Abstract B.
Your objective is to determine which abstract you believe is written by an AI and which one is written by a neurologist.

We kindly ask to do not read the introduction and references before answering the questions.

**Abstract A**

**Introduction** Canine scent detection is a promising method for the rapid and accurate detection of human COVID-19 infections. However, little is known about the performance metrics and training strategies for scent detection dogs. In this study, we aimed to compare the performance of scent detection dogs trained with different sample types and training strategies, and to evaluate the effect of training duration on detection accuracy.

**Methods** We trained ten scent detection dogs using positive and negative sweat samples from 50 RT-PCR-confirmed COVID-19 cases. The dogs were trained using positive reinforcement and clicker training techniques, with training durations ranging from four to eight weeks. We evaluated the dogs' detection accuracy using sweat, saliva, and breath samples collected from 300 volunteers. The samples were analyzed using RT-PCR as the gold standard, and the dogs' performance was assessed using sensitivity, specificity, positive predictive value (PPV), negative predictive value (NPV), and receiver operating characteristic (ROC) curve analysis.

**Results** Our study revealed that scent detection dogs were highly accurate in detecting COVID-19 using all three sample types, with the highest sensitivity and specificity observed for breath samples (sensitivity: 95.7%, specificity: 96.5%). ROC curve analysis showed excellent discriminatory power, with the breath samples having an area under the curve (AUC) of 0.982 (95% confidence interval: 0.968-0.996). We observed variability in detection accuracy among different dog breeds, with Border Collies showing the highest sensitivity and specificity. Training duration was a significant factor affecting detection accuracy, with dogs trained for eight weeks exhibiting higher sensitivity and NPV than those trained for four weeks.

**Conclusion** Our findings demonstrate the potential of scent detection dogs as a rapid and accurate screening tool for human COVID-19 infections, with breath samples providing the highest detection accuracy. Training duration and breed selection are critical factors influencing detection accuracy, with longer training periods associated with higher sensitivity and NPV. Further research is needed to optimize the training and selection of scent detection dogs and to explore the feasibility of using dogs in large-scale COVID-19 screening programs.

**Abstract B**

**Introduction** Previous research demonstrated that medical scent detection dogs have the ability to distinguish SARS-CoV-2 positive from negative samples with high diagnostic accuracy. To deploy these dogs as a reliable screening method, it is mandatory to examine if canines maintain their high diagnostic accuracy in real-life screening settings. We conducted a study to evaluate the performance of medical scent detection dogs under real-life circumstances.

**Methods** Eight dogs were trained to detect SARS-CoV-2 RT-qPCR-positive samples. Four concerts with a total of 2802 participants were held to evaluate canines’ performance in screening individuals for SARS-CoV-2 infection. Sweat samples were taken from all participants and presented in a line-up setting. In addition, every participant had been tested with a SARS-CoV-2 specific rapid antigen test and a RT-qPCR and they provided information regarding age, sex, vaccination status and medical disease history. The participants’ infection status was unknown at the time of canine testing. Safety measures such as mask wearing and distance keeping were ensured.

**Results** The SARS-CoV-2 detection dogs achieved a diagnostic specificity of 99.93% (95% CI 99.74% to 99.99%) and a sensitivity of 81.58% (95% CI 66.58% to 90.78%), respectively. The overall rate of concordant results was 99.68%. The majority of the study population was vaccinated with varying vaccines and vaccination schemes, while several participants had chronic diseases and were under chronic medication. This did not influence dogs’ decisions.

**Conclusion** Our results demonstrate that SARS-CoV-2 scent detection dogs achieved high diagnostic accuracy in a real-life scenario. The vaccination status, previous SARS-CoV-2 infection, chronic disease and medication of the participants did not influence the performance of the dogs in detecting the acute infection. This indicates that dogs provide a fast and reliable screening option for public events in which high-throughput screening is required.

**Task 2: Abstract Identification**

Indicate which abstract you believe is written by AI by selecting one of the options below:

1. Abstract A
2. Abstract
3. I do not know

Please provide a brief explanation, outlining the reasons behind your choice and consider highlighting key elements or characteristics that influenced your judgment. Ensure you have completed Task 2 before proceeding to the next step.

**Task 3: Read Introduction and References**

Read the introduction and references sections for the corresponding abstracts.
If you have not answered the questions in Task 2, please do so before proceeding to read the introduction and references.

**Introduction A**

The COVID-19 pandemic has posed a significant challenge for global healthcare systems, affecting millions of people worldwide. To combat the spread of the virus and safeguard public health, researchers and public health officials have explored various screening methods to rapidly and accurately identify infected individuals. One widely used approach is nucleic acid amplification tests, such as RT-PCR, which detect viral RNA in respiratory samples. While these tests are highly sensitive and specific, they are resource-intensive and require specialized equipment and expertise, limiting their scalability for large-scale screening programs. Other screening methods, such as serological tests that detect antibodies and antigen tests that detect viral proteins, have been explored. Although less resource-intensive, these tests may be less sensitive and specific, potentially missing early-stage infections and asymptomatic cases. An alternative approach showing promise is canine scent detection. Trained dogs have demonstrated high accuracy in detecting COVID-19 using various sample types like sweat, saliva, and breath. Canine scent detection offers advantages such as affordability and scalability, making it particularly useful in settings with limited access to specialized equipment or expertise. Despite its potential, there is still much to uncover about the practical applications and performance of canine scent detection for COVID-19. Further research is needed to optimize the training and selection of scent detection dogs, as well as evaluate the feasibility of implementing this approach in large-scale screening programs.

Therefore, this study aims to assess the performance of scent detection dogs in identifying human COVID-19 infections. It will compare the accuracy of dogs trained with different sample types and training strategies. The findings will have important implications for using scent detection dogs in large-scale COVID-19 screening programs, offering valuable insights into complementing existing methods and enhancing public health efforts in the ongoing battle against the pandemic.

**References A**

1. Adams, L., Johnson, T., Smith, J., et al. (2017). Canine Scent Detection of SARS-CoV-2 Infections in a Field Study. Journal of Veterinary Medicine, 2017;11:ad345.

2. Lee, J., Kim, T., Garcia, M., et al. (2010). Comparing the Accuracy of Canine Scent Detection and Rapid Antigen Tests for COVID-19 Screening. Journal of Infectious Diseases, 2010;7:pn890.

3. Davis, K., Brown, M., Johnson, E., et al. (2012). Exploring the Use of Canine Scent Detection in a COVID-19 Outbreak at a University Campus. Journal of Epidemiology and Community Health, 2012;13:rd234.

4. Perez, B., Lopez, A., Thompson, D., et al. (2016). Canine Scent Detection of SARS-CoV-2 Infections in Healthcare Workers. Journal of Clinical Microbiology, 2016;9:wt678.

5. Green, S., Martinez, R., Brown, J., et al. (2019). Training and Testing Canine Scent Detection for the Diagnosis of COVID-19. Journal of Comparative Psychology, 2019;10:jq901.

6. Davis, R., Miller, J., Lee, B., et al. (2014). Canine Scent Detection of Asymptomatic COVID-19 Cases in Community Settings. Journal of Animal Behavior, 2014;8:zt012.

7. Hernandez, T., Adams, L., Lewis, K., et al. (2022). Canine Scent Detection for Mass Screening of COVID-19 in Prisons. Journal of Correctional Health Care, 2022;12:fg345.

8. Taylor, M., Williams, J., Thompson, E., et al. (2015). A Comparative Study of Canine Scent Detection and RT-PCR for COVID-19 Diagnosis in a Hospital Setting. Journal of Public Health Management and Practice, 2015;14:hi789.

9. Wilson, K., Brown, D., Kim, S., et al. (2018). Canine Scent Detection for Screening of COVID-19 in Homeless Populations. Journal of Occupational and Environmental Medicine, 2018;15:wx567.

10. Lopez, J., Johnson, T., Patel, R., et al. (2011). Comparison of Canine Scent Detection and Serological Tests for COVID-19 in Long-Term Care Facilities. Journal of the American Geriatrics Society, 2011;16:bc456.

**Introduction B**

The use of rapid point of care antigen-tests (PoC-antigen tests) for the diagnosis of a SARS-CoV-2 infection continues to be a key tool to manage the ongoing COVID-19 pandemic. Several studies have shown the ability of medical scent detection dogs to identify samples from SARS-CoV-2 infected individuals with high accuracy, highlighting the role such dogs could play in the management of a pandemic. Previous research showed that different body fluids, such as saliva, sweat and urine and other sample types like worn face masks are suitable for detection, which suggests that there is a general SARS-CoV-2 infection associated odour that dogs can be trained on. In addition, our group demonstrated that such dogs were able to differentiate SARS-CoV-2 infection from other acute viral respiratory tract infections. However, most of the current data were generated in laboratory settings, rather than in a real-world scenario or lacked the verification of SARS-CoV-2 infections by a PoC-antigen test and real-time quantitative reverse transcription-PCR (RT-qPCR). In some countries, SARS-CoV-2 detection dogs were already deployed in pilot projects in public transports, such as airports or underground transportation. In a first real-life trial in an airport setting with samples from 303 passengers, dogs identification and RT-PCR results matched for 97.7% of samples from passengers. Although this study clearly indicated that medical scent detection dogs can maintain their diagnostic accuracy under real-life conditions, the study cohort was too small for far-reaching conclusions. Furthermore, additional field settings should improve the general feasibility of the use of medical detection dogs to demonstrate its diagnostic use in different populations.

The WHO and the Paul Ehrlich Institute (PEI) recommend thresholds for diagnostic specificities and sensitivities for PoC-antigen tests of more than 97% and more than 80%, respectively.To meet the requirements of a valuable diagnostic tool, medical scent detection dogs should also meet these diagnostic performance characteristics, even under real-life circumstances when many distractions could influence their performance.

**References B**

1. Guest C, Dewhirst SY, Lindsay SW, et al. Using trained dogs and organic semi-conducting sensors to identify asymptomatic and mild SARS-CoV-2 infections: an observational study. J Travel Med 2022;29:taac043.

2. Eskandari E, Ahmadi Marzaleh M, Roudgari H, et al. Sniffer dogs as a screening/diagnostic tool for COVID-19: a proof of concept study. BMC Infect Dis 2021;21:243.

3. Vesga O, Agudelo M, Valencia-Jaramillo AF, et al. Highly sensitive scent-detection of COVID-19 patients in vivo by trained dogs. PLoS One 2021;16:e0257474.

4. Grandjean D, Sarkis R, Lecoq-Julien C, et al. Can the detection dog alert on COVID-19 positive persons by sniffing axillary sweat samples? A proof-of-concept study. PLoS One 2020;15:e0243122.

5. Maia RdeCC, Alves LC, da Silva JES, et al. Canine olfactory detection of SARS-COV2-Infected patients: a one health approach. Front Public Health 2021;9:647903.

6. Sarkis R, Lichaa A, Mjaess G, et al. New method of screening for COVID-19 disease using sniffer dogs and scents from axillary sweat samples. J Public Health 2022;44:e36–41.

7. Angeletti S, Travaglino F, Spoto S, et al. COVID-19 sniffer dog experimental training: which protocol and which implications for reliable sidentification? J Med Virol 2021;93:5924–30.

8. Jendrny P, Schulz C, Twele F, et al. Scent dog identification of samples from COVID-19 patients - a pilot study. BMC Infect Dis2020;20:536.

9. Chaber A, Hazel S, Matthews B. Evaluation of canine detection of COVID‐19 infected individuals under controlled settings. Transbound Emerg Dis 2022:1–8.

10. Hag-Ali M, AlShamsi AS, Boeijen L, et al. The detection dogs test is more sensitive than real-time PCR in screening for SARS-CoV-2. Commun Biol 2021;4:686.

**Task 4: Final Assessment**

Based on the complete information, including the introductions and reference sections, make a final assessment by indicating which manuscript you believe was written by AI?

1. Manuscript A
2. Manuscript B
3. I do not know

Please briefly explain why you made your choice and mention any important factors that influenced your decision.

**Thank you for your participation!**

**TEST 2**

**Task 1: Read the Abstracts**

Please carefully read two abstracts labeled as Abstract A and Abstract B.
Your objective is to determine which abstract you believe is written by an AI and which one is written by a neurologist.

We kindly ask to do not read the introduction and references before answering the questions.

**Abstract A**

Steroid responsive meningitis arteritis (SRMA) is an aberrant Th2-mediated systemic

inflammatory disease in dogs. The etiopathogenesis still remains unclear as no triggering pathogen or autoantigen could be found so far. **Hypothesis.** Large high-density peptide microarrays are a suitable screening method to detect possible autoantigens which might be involved in the pathogenesis of SRMA.**Methods.** The IgA and IgG profile of pooled serum samples of 5 dogs with SRMA and 5 dogs with neck pain due to intervertebral disc herniation (IVDH) without ataxia or paresis were compared via commercially available highdensity peptide microarrays (Discovery Microarray) containing 29,240 random linear peptides. Canine distemper virus nucleoprotein (CDVN) served as positive control as all dogs were vaccinated. Common motifs were compared to amino acid sequences of known proteins via databank search. One suitable protein was manually selected for further analysis with a smaller customized high-density peptide microarray. **Results.** Pooled serum of dogs with SRMA and IVDH showed different IgA and IgG responses on Discovery Microarray. Only top IgG responses of dogs with SRMA showed a common motif not related to the control protein CDVN. This common motif is part of the interleukin 1 receptor antagonist protein (IL1Ra). On IL1Ra, dogs with SRMA displayed IgA binding to an additional epitope, which dogs with IVDH did not show. **Discussion.** IL1Ra is an anti-inflammatory acute phase protein. Different immunoglobulin binding patterns on IL1Ra could be involved in the pathogenesis of SRMA and IL1Ra might be developed as future biomarker for SRMA.

**Abstract B**

Steroid-responsive meningitis-arteritis (SRMA) is a relatively common inflammatory disease in dogs that can cause severe neurological deficits and is potentially life-threatening. Diagnosis of SRMA is challenging, with current diagnostic methods lacking sensitivity and specificity. **Hypothesis.** Peptide microarrays could be used to identify potential immunoglobulin biomarkers for SRMA in dogs. **Methods.** We designed a custom peptide microarray containing 120 peptides derived from proteins previously implicated in immune and inflammatory responses. Serum samples were collected from a cohort of 50 dogs diagnosed with SRMA and 50 healthy control dogs. Peptide microarrays were used to profile IgG and IgM responses to the 120 peptides. The identified candidate peptides were validated by performing ELISA assays on a larger cohort of 100 SRMA dogs and 100 healthy control dogs. The incidence and prevalence of SRMA in the population of dogs included in this study were estimated using data from veterinary hospitals and clinics. **Results.** Peptide microarrays revealed that three peptides (GHSR, JCJ, and LR3) showed significantly increased binding to IgG and IgM antibodies in SRMA dogs compared to controls (p < 0.001). GHSR showed the highest specificity and sensitivity (96% and 98%, respectively), followed by JCJ and LR3. ELISA assays confirmed the differential binding of GHSR, JCJ, and LR3 to SRMA sera compared to control sera. The estimated incidence and prevalence of SRMA in the population of dogs included in this study were 1 in 300 and 0.33%, respectively. **Discussion.** Our study provides evidence that peptide microarrays can be used to identify potential immunoglobulin biomarkers for SRMA in dogs. The identified candidate peptides, GHSR, JCJ, and LR3, show promise as diagnostic tools for SRMA in future studies. GHSR, in particular, shows high specificity and sensitivity, making it a promising candidate for further investigation. The estimated incidence and prevalence of SRMA in our study provide valuable epidemiological data that can aid in the understanding and management of this disease in dogs. Our results suggest that peptide microarrays have the potential to be used as a non-invasive, sensitive, and specific diagnostic tool for SRMA in dogs. Further studies are needed to confirm these findings in larger populations and to evaluate the clinical utility of these biomarkers. Our study represents an important step towards the development of improved diagnostic tools for SRMA, which could ultimately lead to better outcomes for affected dogs.

**Task 2: Abstract Identification**

Indicate which abstract you believe is written by AI by selecting one of the options below:

1. Abstract A
2. Abstract
3. I do not know

Please provide a brief explanation, outlining the reasons behind your choice and consider highlighting key elements or characteristics that influenced your judgment. Ensure you have completed Task 2 before proceeding to the next step.

**Task 3: Read Introduction and References**

Read the introduction and references sections for the corresponding abstracts.
If you have not answered the questions in Task 2, please do so before proceeding to read the introduction and references.

**Introduction A**

Steroid-responsive meningitis-arteritis (SRMA) is an inflammatory disease in dogs mostly affecting the meninges and meningeal arteries of the neck. Affected dogs are mostly young-adult and show signs of neck pain, fever, blood leukocytosis, and increased protein and neutrophilic pleocytosis in the cerebrospinal fluid (CSF) as well as a systemic acute phase response with increased C-reactive protein and serum amyloid-A in the serum. Increased levels of immunoglobulin (Ig)A can be found in serum and CSF. The clinical diagnosis is achieved by combining typical clinical signs and laboratory findings after exclusion of an infectious agent. The etiopathogenesis is not fully understood, yet. It is suspected that an unknown trigger causes an aberrant Th2-mediated inflammation in susceptible individuals. Several studies have failed to reveal the initial trigger so far. Therefore, the assumption prevails that after fast elimination of the presumed triggering antigen the altered immune system remains active to keep the harmful inflammation. In human medicine, molecular mimicry is one known pathomechanism for inducing autoimmune diseases. Knowing the responsible triggering antigen or the equivalent cross-reacting host protein might be fundamental to completely understand the pathomechanism of SRMA and develop adequate therapy or even prevent the disease.

As a direct search for triggering antigens using various methods has so far been unsuccessful, search for immunoglobulins indicating a former host-antigen contact might be useful: Immunoglobulins formed against pathogens in the course of an infection can still be detected in the blood longtime after the actual infection and are very specific in their binding capacity. Searching for immunoglobulins required knowing which triggering antigen is responsible for the disease. However, using the novel technology of high-density microarrays, such knowledge is no longer necessary to find the antibodies involved. High-density microarrays allow for untargeted screening for immunoglobulin patterns. Each mammal develops its own highly individualized immunoglobulin profile due to individual antigen exposure during its lifetime. These profiles overlap in patients with the same disease. Overlaps could be visualized in studies using microarrays and can be used to either diagnose the disease or to help track down the presumed triggering antigen.

High-density microarrays are glass plates very tightly printed with different peptides. Approximately 35,000 different peptides fit on one glass plate. Binding of immunoglobulins to the respective peptides can be made quantifiable by fluorescence staining techniques. In this way, an exceptionally large number of possible binding possibilities can be investigated within a short time using a small sample quantity, without having to know the antibody binding epitope of the unknown antigen in advance. In a second step, it is possible to further classify the antigen structure via more precise epitope searches and with the comparison of known proteins of antigens in existing databases, and thus to track down the antigen or the pathogen.

This technology just starts to receive attention in veterinary medicine. Initial steps included for example characterizing the immunoglobulin profile in canine lymphoma. Lake et al. proved that the immunoglobulin profile of dogs with meningoencephalitis of unknown origin differ from that of healthy dogs and dogs with brain tumor, each with a sensitivity and specificity of 100%, but a more detailed classification was not performed.

The following study is a pilot study to screen for possible immunoglobulin profiles in dogs with SRMA which could be used to further explore a potential candidate protein acting as autoantigen in dogs with SRMA. The detection of such a profile could help to further shed light on the etiopathogenesis of the disease and could be used as diagnostic or prognostic biomarker in SRMA.

**References A**

1. Bathen-Noethen A, Carlson R, Menzel D, Mischke R, Tipold A. Concentrations of acute-phase proteins in dogs with steroid responsive meningitis-arteritis. J Vet Intern Med. 2008 Sep-Oct; 22(5):1149–56. https://doi.org/10.1111/j.1939-1676.2008.0164.x PMID: 18691368

2. Lowrie M, Penderis J, Eckersall PD, McLaughlin M, Mellor D, Anderson TJ. The role of acute phase proteins in diagnosis and management of steroid-responsive meningitis arteritis in dogs. Vet J. 2009 Oct;182(1):125–30. https://doi.org/10.1016/j.tvjl.2008.05.001 PMID: 18590973

3. Rose JH, Harcourt-Brown TR. Screening diagnostics to identify triggers in 21 cases of steroid-responsive meningitis-arteritis. J Small Anim Pract. 2013 Nov; 54(11):575–8. https://doi.org/10.1111/jsap.12143 PMID: 24580013

4. Pfister K, Silaghi C, Kohn B, Lübke-Becker A, Mietze A, Pfeffer M, et al. Testing for Vector Transmitted Microorganisms in Dogs with Meningitis and Meningoencephalitis of Unknown Aetiology. J Vet Med Res. 2015; 2(1): 1014. https://doi.org/10.47739/2378-931X/1014

5. Bailey Timothy L., Johnson James, Grant Charles E., Noble William S., "The MEME Suite", Nucleic Acids Research, 43(W1):W39–W49, 2015 https://doi.org/10.1093/nar/gkv416 PMID: 25953851

6. Scarisbrick IA, Rodriguez M. Hit-Hit and hit-Run: viruses in the playing field of multiple sclerosis. Curr Neurol Neurosci Rep. 2003 May; 3(3):265–71. https://doi.org/10.1007/s11910-003-0087-9 PMID:12760396

7. Shahrizaila N, Yuki N. Guillain-barre´ syndrome animal model: the first proof of molecular mimicry in human autoimmune disorder. J Biomed Biotechnol. 2011; 2011:829129. https://doi.org/10.1155/2011/829129 PMID: 21197269

8. Cusick MF, Libbey JE, Fujinami RS. Molecular mimicry as a mechanism of autoimmune disease. ClinRev Allergy Immunol. 2012 Feb; 42(1):102–11. https://doi.org/10.1007/s12016-011-8294-7 PMID: 22095454

9. Tizard IR. Veterinary Immunology-E-Book. Amsterdam: Elsevier Health Sciences, 2017.

10. Breitling F, Nesterov A, Stadler V, Felgenhauer T, Bischoff FR. High-density peptide arrays. Mol Biosyst. 2009 Mar; 5(3):224–34. https://doi.org/10.1039/b819850k PMID: 19225611

**Introduction B**

Steroid-responsive meningitis-arteritis (SRMA) is a relatively common inflammatory disease in dogs that affects the meninges and blood vessels, leading to severe neurological deficits and potential life-threatening complications. Accurate and timely diagnosis of SRMA is crucial for effective treatment and management of affected dogs. However, current diagnostic methods for SRMA lack the desired sensitivity and specificity, posing challenges for clinicians in accurately identifying and monitoring this complex disease.

In recent years, significant advancements have been made in the field of biomarker discovery for various diseases, including inflammatory conditions. Peptide microarrays have emerged as a powerful tool for high-throughput screening of immunoglobulin profiles and have shown promise in identifying potential biomarkers associated with different pathologies. Therefore, based on our abstract, we propose utilizing large high-density peptide microarrays as a screening method to detect candidate proteins, such as GHSR, JCJ, and LR3, for future biomarker detection in dogs with SRMA.

The motivation behind this research stems from the urgent need for improved diagnostic tools that can aid in the early detection, accurate diagnosis, and monitoring of SRMA in dogs. The identification of specific biomarkers for SRMA would not only enhance our understanding of the underlying immunological mechanisms but also facilitate the development of targeted therapeutic interventions.

To establish the validity and efficacy of large high-density peptide microarrays for immunoglobulin profiling in dogs with SRMA, we draw upon several plausible research studies conducted in the field of peptide microarray-based biomarker discovery. For instance, a study by Smith et al. demonstrated the successful application of peptide microarrays in identifying novel autoantibodies associated with autoimmune disorders in humans. Similarly, Johnson et al. utilized peptide microarrays to discover potential biomarkers for a canine-specific autoimmune disease, showing promising results in terms of sensitivity and specificity.

In our study design, we propose the use of a custom-designed peptide microarray containing a comprehensive repertoire of peptides derived from proteins that have previously been implicated in immune and inflammatory responses, including GHSR, JCJ, and LR3. By profiling the IgG and IgM responses to these peptides in serum samples from a cohort of 100 dogs diagnosed with SRMA and 100 healthy control dogs, we aim to identify candidate peptides that exhibit statistically significant differential binding to antibodies in SRMA dogs compared to controls (p < 0.001).

Moreover, to validate the findings from the microarray analysis, we plan to perform enzyme-linked immunosorbent assay (ELISA) assays on an extended cohort of 500 SRMA dogs and 500 healthy control dogs. This validation step will provide further evidence of the differential binding of the identified candidate peptides, such as GHSR, JCJ, and LR3, to SRMA sera, establishing their potential as diagnostic tools for SRMA with a specificity of 96% and sensitivity of 98%.

In addition to the discovery of candidate peptides, our study aims to estimate the incidence and prevalence of SRMA in the population of dogs included in the investigation. By analyzing data from a nationwide survey of veterinary hospitals and clinics, we will estimate the incidence of SRMA in the dog population to be approximately 1 in 300, with a prevalence of 0.33%.

In conclusion, based on our abstract, our study proposes the utilization of large high-density peptide microarrays as a screening method for immunoglobulin profiling in dogs with SRMA. By leveraging the potential of this innovative approach, we aim to identify candidate proteins, including GHSR, JCJ, and LR3, that exhibit statistically significant differential binding to antibodies in SRMA dogs, thereby paving the way for the development of improved diagnostic tools for this complex disease. Furthermore, the estimation of SRMA incidence and prevalence will provide important epidemiological insights that can enhance our knowledge and management of SRMA in the dog population. Through our research, we hope to contribute to the advancement of veterinary medicine and ultimately improve the lives of dogs affected by SRMA.

**References B**

1. Johnson R, Smith C, Thompson E. Advances in Peptide Microarray-Based Biomarker Discovery. J Immunol Res. 2005; 12(3):456-467. doi:10.1155/2005/123456. PMID: 12345678

2. Davis P, Wilson M, Brown K. Novel Autoantibodies Identified Using Peptide Microarrays in Autoimmune Disorders. J Clin Immunol. 2010; 123(4):567-578. doi:10.1007/s10875-2010-1234-5678. PMID: 23456789

3. Smith J, Johnson D, Anderson L. Peptide Microarray Analysis Reveals Canine-Specific Autoantibodies in an Autoimmune Disease Model. Vet Immunol Immunopathol. 2015; 234(2):89-98. doi:10.1016/j.vetimm.2015.07.006. PMID: 34567890

4. Thompson S, Brown R, Taylor J. Immunoglobulin Profiling with High-Density Peptide Microarrays: An Innovative Approach for Biomarker Discovery. Mol Immunol. 2018; 987(5):321-334. doi:10.1016/j.molimm.2018.08.009. PMID: 45678901

5. Harris E, Martinez M, Patel R. Evaluating the Sensitivity and Specificity of Peptide Microarrays for Immunoglobulin Profiling in Canine Diseases. Vet Diagn Invest. 2020; 876(1):12-22. doi:10.1177/1234567890123456. PMID: 56789012

6. Roberts T, Jackson P, Garcia M. Epidemiological Insights on Steroid-Responsive Meningitis-Arteritis in Dogs: A Nationwide Survey. Vet J. 2017; 654(3):234-245. doi:10.1016/j.tvjl.2017.11.012. PMID: 67890123

7. Lee H, Davis S, Wilson K. Diagnostic Challenges and Advancements in Steroid-Responsive Meningitis-Arteritis: A Comprehensive Review. Vet Pathol. 2019; 987(4):98-112. doi:10.1177/2345678901234567. PMID: 78901234

8. Martinez G, Thompson R, Brown L. Peptide Microarray Profiling of Immunoglobulin Subclasses in Canine Inflammatory Diseases. J Vet Res. 2021; 432(2):56-67. doi:10.1002/jvr.2021.432.056. PMID: 89012345

9. Wilson M, Johnson K, Anderson L. High-Density Peptide Microarrays: A Promising Tool for Immunoglobulin Biomarker Discovery in Canine Diseases. Vet Immunol. 2018; 876(2):78-89. doi:10.1016/j.vetimm.2018.03.004. PMID: 90123456

10.Garcia A, Taylor M, Roberts E. Peptide Microarray-Based Profiling of Immunoglobulin Responses in Canine Steroid-Responsive Meningitis-Arteritis. J Vet Med Sci. 2016; 123(6):789-800. doi:10.1292/jvms.16-0123. PMID: 12345678

**Task 4: Final Assessment**

Based on the complete information, including the introductions and reference sections, make a final assessment by indicating which manuscript you believe was written by AI?

1. Manuscript A
2. Manuscript B
3. I do not know

Please briefly explain why you made your choice and mention any important factors that influenced your decision.

**Thank you for your participation!**

**TEST 3**

**Task 1: Read the Abstracts**

Please carefully read two abstracts labeled as Abstract A and Abstract B.
Your objective is to determine which abstract you believe is written by an AI and which one is written by a neurologist.

We kindly ask to do not read the introduction and references before answering the questions.

**Abstract A**

Epilepsy is a common neurological disorder in dogs, affecting approximately 1% of the population. The endocannabinoid system has been implicated in the pathogenesis of epilepsy, and the cannabinoid receptor type 1 (CB1) has been identified as a potential therapeutic target. The hippocampus is a brain region that plays a crucial role in the development and propagation of seizures, and it has been suggested that alterations in CB1 expression in this region may contribute to the development of epilepsy. In this study, we examined the hippocampal expression of CB1 in dogs with epilepsy compared to healthy controls. We analyzed hippocampal tissue samples from 30 dogs with epilepsy and 20 healthy controls using quantitative real-time PCR (qPCR). Our results showed a significant decrease in CB1 expression in the hippocampus of dogs with epilepsy compared to controls (p < 0.05). We also found a significant correlation between CB1 expression and seizure frequency (r = -0.57, p < 0.01), with lower CB1 expression associated with higher seizure frequency. Furthermore, we investigated the effect of antiepileptic medication on CB1 expression in the hippocampus of dogs with epilepsy. We compared hippocampal tissue samples from 15 dogs with epilepsy receiving antiepileptic medication to 15 dogs with epilepsy not receiving medication. Our results showed no significant difference in CB1 expression between the two groups (p > 0.05), suggesting that medication does not significantly affect CB1 expression in the hippocampus. Overall, our findings suggest that decreased CB1 expression in the hippocampus may contribute to the development and progression of epilepsy in dogs. Targeting CB1 in the treatment of canine epilepsy may therefore hold promise as a therapeutic strategy.

**Abstract B**

Canine drug-resistant epilepsy is a prevailing issue in veterinary neurology. Alternative or additional treatment with cannabinoids is showing promising results in seizure management. A crucial component of the endocannabinoid system, cannabinoid receptor type 1 (CB1R), is heavily involved in the control of neurotransmitter release. Knowledge of its distribution in the epileptic brain would serve a better understanding of disease pathology and application of cannabinoids in dogs with epilepsy. CB1R distribution was assessed in sub-regions of hippocampus of dogs with idiopathic epilepsy, structural epilepsy and without cerebral pathology. In dogs with idiopathic epilepsy, significantly decreased CB1R expression compared to control animals was observed in CA1. In dogs with structural epilepsy, a significant increase in CB1R signal intensity in comparison to controls was observed. CB1R expression was higher in the structural group as compared to the idiopathic. Double immunofluorescence showed co-localization between CB1R and an astrocytic marker in about 50% of cells, regardless of the diagnosis. In summary, CB1R expression in canine hippocampus undergoes modification by the epileptic process and the direction of this change depends on the etiology of the disease. The distinct disease-associated CB1R expression needs to be considered in new treatment development for dogs with epilepsy.

**Task 2: Abstract Identification**

Indicate which abstract you believe is written by AI by selecting one of the options below:

1. Abstract A
2. Abstract B
3. I do not know

Please provide a brief explanation, outlining the reasons behind your choice and consider highlighting key elements or characteristics that influenced your judgment. Ensure you have completed Task 2 before proceeding to the next step.

**Task 3: Read Introduction and References**

Read the introduction and references sections for the corresponding abstracts.
If you have not answered the questions in Task 2, please do so before proceeding to read the introduction and references.

**Introduction A**

Epilepsy is a prevalent and complex neurological disorder characterized by recurring seizures, affecting not only humans but also a significant number of dogs worldwide. Despite extensive research efforts, the underlying mechanisms of epilepsy in dogs remain incompletely understood, necessitating further investigation to advance our understanding of the disease and develop more effective treatment strategies. In recent years, the endocannabinoid system has emerged as a promising avenue of research in the field of epilepsy, with particular emphasis on the cannabinoid receptor type 1 (CB1) and its potential role in the pathogenesis of the disorder.

The endocannabinoid system comprises a complex network of endogenous cannabinoids, receptors, and enzymes involved in regulating various physiological processes, including neuronal excitability, synaptic plasticity, and neuroinflammation. CB1, a G protein-coupled receptor primarily expressed in the central nervous system, plays a crucial role in modulating neurotransmission and maintaining the balance of neuronal activity. Dysregulation of CB1 signaling has been implicated in several neurological disorders, including epilepsy.

The hippocampus, a vital brain region known for its involvement in learning, memory, and seizure generation, has been a subject of significant interest in epilepsy research. Alterations in hippocampal structure and function have been observed in both human and animal models of epilepsy, suggesting its critical role in the pathophysiology of the disorder. Moreover, studies have indicated that changes in CB1 expression within the hippocampus may contribute to the development and progression of epilepsy, highlighting the importance of investigating CB1 as a potential target in epilepsy research.

Although considerable progress has been made in understanding the role of CB1 in epilepsy, a significant research gap exists concerning its implications specifically in the context of canine epilepsy. Canine models of epilepsy offer a unique opportunity to study the disease in a naturally occurring setting, closely mimicking the complexity and heterogeneity of human epilepsy. By addressing this research gap, we aim to provide valuable insights into the involvement of CB1 in the pathophysiology of canine epilepsy, contributing to our understanding of epilepsy across species.

This study aims to meticulously examine the expression patterns of CB1 within the hippocampus of dogs diagnosed with epilepsy, utilizing a well-defined cohort of affected animals alongside an age-matched control group of healthy dogs. To assess CB1 expression levels, we will employ quantitative real-time PCR (qPCR), a highly accurate and sensitive molecular technique widely used for gene expression analysis. By comparing CB1 expression between epileptic dogs and healthy controls, we seek to determine whether significant differences exist, elucidating the potential role of CB1 in the development and progression of canine epilepsy.

Furthermore, we will explore potential correlations between CB1 expression levels and seizure frequency in the epilepsy cohort, aiming to establish a link between CB1 dysregulation and the clinical manifestation of the disease. Additionally, we will investigate the impact of antiepileptic medications on CB1 expression within the hippocampus by comparing tissue samples from dogs receiving medication to those without medication, providing insights into the influence of treatment on CB1 dynamics.

Through this comprehensive analysis, our study aims to advance our understanding of the intricate pathogenesis of canine epilepsy and shed light on the potential therapeutic implications of CB1 modulation. The findings may not only contribute to the growing body of knowledge in epilepsy research but also pave the way for the development of targeted therapeutic strategies for the management of this debilitating neurological disorder.

In conclusion, investigating the involvement of CB1 in canine epilepsy represents a significant step toward deciphering the complex mechanisms underlying the disease. By studying CB1 expression within the hippocampus and exploring its correlations with seizure frequency and antiepileptic medication use, we aim to enhance our understanding of canine epilepsy

**References A**

1. Smith, J. R., et al. The role of the endocannabinoid system in the pathogenesis of canine epilepsy. J. Neurosci. Res. 45(3), 234-242 (1995).

2. Johnson, A. L., et al. Hippocampal alterations in CB1 receptor expression in a rat model of temporal lobe epilepsy. Neurosci. Lett. 78(2), 167-173 (1997).

3. Thompson, G. A., et al. Excitatory and inhibitory synaptic dysregulation in the hippocampus of dogs with epilepsy. Brain Res. 1122(1), 189-198 (2001).

4. Davis, M. B., et al. Genetic factors influencing CB1 receptor expression in canine epilepsy. Mol. Genet. Genomics. 82(5), 421-429 (2004).

5. Rodriguez-Gomez, J. A., et al. Altered expression of endocannabinoid-related genes in the hippocampus of dogs with epilepsy. Epilepsy Res. 96(1-2), 96-104 (2006).

6. Martinez, C. M., et al. Epileptogenesis in canine models: insights into the molecular mechanisms. J. Neurochem. 70(6), 2570-2577 (2009).

7. Adams, L. A., et al. The impact of CB1 receptor modulation on seizure susceptibility in canine epilepsy. Epilepsy Behav. 81, 87-94 (2011).

8. Garcia-Solis, P., et al. Neuroinflammation and epileptogenesis: insights from canine models. Front. Vet. Sci. 5, 109 (2013).

9. Hernandez-Avalos, I., et al. Abnormal calcium receptor expression in the hippocampus of epileptic dogs. Neurosci. Lett. 124(3), 198-204 (2015).

10. Thompson, M. D., et al. CB1 receptor-mediated signaling in the pathogenesis of canine epilepsy. Neurochem. Res. 136(4), 891-901 (2017).

**Introduction B**

Epilepsy is the most common chronic neurological disease with a lifetime prevalence of 0.76% in the human population and 0.6–0.75% in the total canine population. However, some purebred dog lines show a predisposition to develop epilepsy, which results in higher prevalence ranging from 3 to even 33% in some breeds, posing this disorder as one of the most relevant for veterinary neurology. In addition, high occurrence of epilepsy resistant to treatment with the most commonly used drugs has to be considered, which is estimated to appear in 20–30% of dogs with epilepsy Therefore, it is crucial to develop and validate alternative therapeutic approaches.

Based on therapeutic success in humans, application of hemp-based medicines in dogs started to increase rapidly Delta(9)-tetrahydrocannabinolic acid (Δ9-THC) is a phytocannabinoid present in a hemp plant, which was reported to exert proconvulsant, but also putative anticonvulsant effects. These effects of Δ9-THC are mediated by its action as a partial and/or full agonist of cannabinoid receptor type 1 (CB1R), an important component of the endocannabinoid system (ECS). ECS curbs neuronal excitability through retrograde CB1R signaling, which responds to endocannabinoids synthesized in the postsynaptic terminal in a response to increasing intracellular Ca^2+^levels during action potential. CB1R activation on presynaptic terminals suppresses neurotransmitter release into synaptic cleft predominantly by inhibiting voltage-gated Ca^2+^channels. Thus, hypofunction of any element of the ECS could lead to neuronal hyperexcitability, which may manifest as epileptic seizures.

Studies with mice provided evidence of CB1R as a potential target to stop prolonged seizure activity, delay seizure generalization and shorten duration of status epilepticus. However, modification of target expression could influence therapeutic responses. Thus, it is of particular interest to test whether disease-associated regulation of CB1R needs to be considered. Several studies aimed to describe its redistribution in regions particularly relevant for seizure generation and epileptogenesis, such as hippocampal formation. Analysis of tissue from human patients with temporal lobe epilepsy indicated that while total levels of hippocampal expression of CB1R seem to be decreased there is a selective increase on inhibitory neurons. In rat models of chemically induced epileptogenesis, CB1R expression was enhanced in hippocampus both in the early post-insult phase and the chronic phase with recurrent seizures, indicating the influence of disease development on receptor expression. In mice, severe, frequent seizures were associated with a larger decrease of hippocampal CB1R expression as compared to animals developing milder, less frequent seizures. Thus, dynamics of CB1R expression is closely bound to the course of epilepsy. Their description in canine patients would bring additional insight into disease pathology and therapeutic application of cannabinoids. It is noteworthy to mention that canine epilepsy is typically classified into two types according to the etiology: idiopathic (determined genetically or diagnosed by exclusion of other causes) and structural (originating from forebrain disorders) epilepsy. Even though the two types could be similar in their clinical manifestations, their histopathology and pathophysiology vary and might influence the distribution of CB1R differently. Taking in consideration all of above aspects, the aim of this study was to investigate CB1R expression patterns in the hippocampus of dogs with epilepsy and to qualitatively and quantitatively analyze it with regard to different etiologies of the disease and to test the hypothesis that CB1R expression differs between these two etiologies.

**References B**

1. Beghi, E. Te epidemiology of epilepsy. Neuroepidemiology 54(2), 185–191 (2020).

2. Hulsmeyer, V. I. et al. International veterinary epilepsy task force’s current understanding of idiopathic epilepsy of genetic or suspected genetic origin in purebred dogs. BMC Vet. Res. 11, 175 (2015).

3. Berendt, M., Gullov, C. H. & Fredholm, M. Focal epilepsy in the Belgian shepherd: evidence for simple Mendelian inheritance. J. Small Anim. Pract. 50(12), 655–661 (2009).

4. Tomas, W. B. Idiopathic epilepsy in dogs and cats. Vet. Clin. N. Am. Small Anim. Pract. 40(1), 161–179 (2010).

5. Coelho, R. C. et al. Current review of hemp-based medicines in dogs. J. Vet. Pharmacol. Terap. 44(6), 870–882 (2021).

6. Schep, L. J. et al. Te clinical toxicology of cannabis. N. Z. Med. J. 133(1523), 96–103 (2020).

7. Devinsky, O. et al. Cannabidiol: Pharmacology and potential therapeutic role in epilepsy and other neuropsychiatric disorders. Epilepsia 55(6), 791–802 (2014).

8. Anderson, L. L. et al. Interactions between cannabidiol and Delta(9) tetrahydrocannabinol in modulating seizure susceptibility and survival in a mouse model of Dravet syndrome. Br. J. Pharmacol. 177(18), 4261–4274 (2020).

9. Benson, M. J. et al. Evaluation of the Possible Anticonvulsant Efect of Delta(9)-Tetrahydrocannabinolic Acid in Murine Seizure Models 12 (Cannabis and Cannabinoid Research, 2020).

10. Paronis, C. A. et al. Δ(9)-Tetrahydrocannabinol acts as a partial agonist/antagonist in mice. Behav. Pharmacol. 23(8), 802–805 (2012).

**Task 4: Final Assessment**

Based on the complete information, including the introductions and reference sections, make a final assessment by indicating which manuscript you believe was written by AI?

1. Manuscript A
2. Manuscript B
3. I do not know

Please briefly explain why you made your choice and mention any important factors that influenced your decision.

**Thank you for your participation!**
